# Rheological properties of cells measured by optical tweezers

**DOI:** 10.1186/s13628-016-0031-4

**Published:** 2016-06-22

**Authors:** Yareni A. Ayala, Bruno Pontes, Diney S. Ether, Luis B. Pires, Glauber R. Araujo, Susana Frases, Luciana F. Romão, Marcos Farina, Vivaldo Moura-Neto, Nathan B. Viana, H. Moysés Nussenzveig

**Affiliations:** LPO-COPEA, Instituto de Ciências Biomédicas, Universidade Federal do Rio de Janeiro, Rio de Janeiro, Rio de Janeiro 21941-902 Brazil; Instituto de Física, Universidade Federal do Rio de Janeiro, Rio de Janeiro, Rio de Janeiro 21941-972 Brazil; Laboratório de Ultraestrutura Celular Hertha Meyer, Instituto de Biofisica Carlos Chagas Filho, Universidade Federal do Rio de Janeiro, Rio de Janeiro, Rio de Janeiro 21941-902 Brazil; Universidade Federal do Rio de Janeiro – Pólo de Xerém, Duque de Caxias, Rio de Janeiro 25245-390 Brazil; Instituto Estadual do Cérebro Paulo Niemeyer, Rio de Janeiro, Rio de Janeiro 20231-092 Brazil

**Keywords:** Rheology, Neurons, Astrocytes, Fibroblasts, Optical tweezers, Cell viscoelasticity, Membrane-cortex complex

## Abstract

**Background:**

The viscoelastic properties of cells have been investigated by a variety of techniques. However, the experimental data reported in literature for viscoelastic moduli differ by up to three orders of magnitude. This has been attributed to differences in techniques and models for cell response as well as to the natural variability of cells.

**Results:**

In this work we develop and apply a new methodology based on optical tweezers to investigate the rheological behavior of fibroblasts, neurons and astrocytes in the frequency range from 1Hz to 35Hz, determining the storage and loss moduli of their membrane-cortex complex. To avoid distortions associated with cell probing techniques, we use a previously developed method that takes into account the influence of under bead cell thickness and bead immersion. These two parameters were carefully measured for the three cell types used. Employing the soft glass rheology model, we obtain the scaling exponent and the Young’s modulus for each cell type. The obtained viscoelastic moduli are in the order of Pa. Among the three cell types, astrocytes have the lowest elastic modulus, while neurons and fibroblasts exhibit a more solid-like behavior.

**Conclusions:**

Although some discrepancies with previous results remain and may be inevitable in view of natural variability, the methodology developed in this work allows us to explore the viscoelastic behavior of the membrane-cortex complex of different cell types as well as to compare their viscous and elastic moduli, obtained under identical and well-defined experimental conditions, relating them to the cell functions.

**Electronic supplementary material:**

The online version of this article (doi:10.1186/s13628-016-0031-4) contains supplementary material, which is available to authorized users.

## Background

Cells in their natural environment are continually subjected to internal and external forces that influence their behavior [1, 2]. It has been shown that the elastic properties of the membrane-cortex complex are related to the cell biological functions [3]. Those properties play important roles in a variety of cell processes like growth, division, migration, differentiation and phagocytosis [4]. The cell cortex is a thin cross-linked actomyosin layer immediately beneath the plasma membrane, to which it is connected by transmembrane proteins [4]. We refer to this ensemble as “membrane-cortex complex” (MCC).

Although the detailed mechanism of those mechano-biological processes is still unclear, it has been demonstrated that cells are able to sense and to adapt to the stiffness of the substrate [5, 6]. It has also been shown that in the course of some diseases like malaria, asthma or arthritis, cells take on a stiffer state [7]. For neuronal cells, stiffening is related to Alzheimer’s disease [8], unlike cancer cells, that tend to soften, in order to facilitate metastasis [9, 10]. Softening is also important for embryonic stem cells, which were shown to be more responsive to the application of small cyclic stresses than when they are in a stiffer and differentiated state [11, 12] and for astrocytes, which tend to soften after a traumatic mechanical injury [13].

The ability of cells to soften and/or stiffen their internal structures relies on the viscoelastic nature of their cytoskeleton [14, 15]. This highly dynamic network of proteins responds to chemical and mechanical signals by reorganizing its molecular structure and changing its properties in response to a stimulatory signal [16, 17]. Hence, modeling and characterizing the viscoelastic properties of the cytoskeleton in qualitative and quantitative ways allows for a better understanding of cell biomechanics and signal transduction.

Rheology measurements have revealed that the cytoskeleton response to external stimuli shows a universal behavior, characterized by a fractal power law dependence with frequency [14, 18, 19]. These measurements show that cells actively respond to stretch [20, 21], leading the cytoskeleton to become more solid-like for a constant stretch and more liquid-like for a transient one [17]. The cytoskeleton can be modeled as an active soft glassy material (SGM) with storage and loss moduli varying according to the stimulus, although with important differences [16, 18, 22]. The structural damping law, according to which the ratio of loss to storage is independent of frequency, is used to interpret this behavior [16, 18, 23].

In the past few years a variety of cell rheology techniques have been developed in order to characterize different cell types under different physiological conditions [10, 16, 24–29]. The results have been investigated by Atomic Force Microscopy (AFM), Optical Tweezers (OT), Magnetic Twisting Cytometry (MTC) and other techniques [20]. OT are a technique similar to MTC in the sense that both are able to exert a unidirectional force parallel to the coverslip, thus producing a lateral displacement combined with a rotation of the probe bead [16, 24, 30]. An advantage of the MTC is that many cells can be probed simultaneously, whereas OT probe a single cell. However, OT allow one to choose the region of the cell to be probed, which is a significant advantage, since the mechanical properties have been found to change according to the cell region [27, 31]. Moreover, OT are also capable of indenting cells, allowing comparisons with AFM [32]. However, the values obtained by each of these different methods can differ by up to two orders of magnitude [27, 31–34], even for the same cell type. These differences arise mainly from the different techniques used to perturb the cell, the mathematical model applied to obtain the viscoelastic values and the model used to characterize the geometrical influence of the cell contact with the force transducer (microspheres for OT and MTC and cantilevers for AFM). Also important are the cell culture conditions, the natural variability among cells, the nature and geometry of the substrate, and the region of the cell where the measurements are performed [27, 31, 35–37]. Altogether these details render very difficult quantitative comparisons between reported data [38–40].

Here we employ an OT to dynamically perturb and measure the viscoelastic MCC response of fibroblasts, neurons and astrocytes, all of which are known to respond to mechanical stimuli [41–43]. We scan the frequency range from 1 to 35 Hz, associated with cell normal metabolic time scales and with linear viscoelasticity. The viscoelastic properties of fibroblasts reported in literature, measured by different techniques, vary from Pa to kPa [25–27, 29, 31, 33, 34, 44]. This large variation is also found for astrocytes and neurons [36, 43, 45–47].

In order to avoid distorting the results by geometrical features of the cell probing technique, we apply a previously developed method that takes into account the influence of under bead cell thickness and bead immersion [30, 48]. This approach allows to explore the viscoelastic behavior of different cell types and to get improved values for their viscous and elastic moduli. The fact that we employ the same technique for all cell types renders significant to draw comparisons among them from the results.

## Methods

### Cell cultures

Three different cell types are employed: NIH3T3 fibroblasts, and primary cultures of cortical neurons and cortical astrocytes.

Astrocytes were obtained from neonatal Swiss mice, by dissociating the cerebral cortex following previously established procedures [49]. Cells were seeded in DMEM-F12 supplemented with L-glutamine, 10 % fetal bovine serum and 1 % penicillin/streptomycin, and allowed to proliferate until confluence.

Neurons were obtained from E14 Swiss mouse embryos, by dissociating the cells from the cerebral cortex and plating them in Neurobasal media supplemented with L-glutamine, 1 % penicillin/streptomycin and 2 % of B27, following previously established procedures [49].

Fibroblast NIH3T3 cells were cultured in DMEM-F12 supplemented with L-glutamine, 10 % fetal bovine serum and 1 % penicillin/streptomycin.

Culture reagents, unless otherwise mentioned, were all purchased from Invitrogen (Carlsbad, CA). Cells were all maintained at 37 °C and 5 % CO_2_.

NIH3T3 fibroblasts and astrocytes were split the day before the experiments using phosphate buffered saline (PBS)/EDTA 0.02 %. Neuronal cell cultures were prepared the day before the experiments. 2×10^5^ cells were plated on a 18 × 18 mm glass coverslip pre-coated with poly-L-lysine and placed within a special 35 mm glass-bottom dish culture plate.

### Optical tweezers setup and calibration

The OT system consists of an infrared Nd:YVO4 Osprey laser (λ = 1064 nm) (Quantronix, USA) that illuminates the back focal plane of a PLAN APO 100X 1.4 NA DIC H Nikon objective lens attached to an inverted Nikon Eclipse Ti-S microscope (Nikon, Melville, NY) to create the optical trap. A homemade temperature/CO_2_ chamber is coupled to the microscope, allowing cells to remain in optimal culture conditions throughout the experiments (37 ± 0.5 °C; 5.0 ± 0.5 % of CO_2_ and pH 7.0 - 7.5). A piezoelectric stage E-710 (PI, Germany) is also coupled to the microscope in order to control the sample position with nanometric precision.

To calibrate the OT, a sample, containing polystyrene beads with radius *a* = (1.52 ± 0.02) μm (Polysciences, Warrington, PA), is placed on the microscope. A single bead is trapped and the sample is set to move with different controlled velocities. Movies of the entire process are recorded and analyzed to obtain the bead position displacements. The trap transverse stiffness per unit power *P* at the objective entrance is *k/P* = (0.12 ± 0.02)pN μm^-1^ mW^-1^. The trapped bead displacement multiplied by the value of the trap stiffness gives the optical force on the bead. The trap stiffness values can be increased or decreased by changing the laser beam power [50].

### MCC rheology

The cytoskeleton is most commonly modelled as a soft glassy material [16, 18]. Its viscoelastic response to an oscillating stimulus of frequency *f* can be described by the so-called “structural damping law” with an additional Newtonian viscous term, given by:

1 $$ G(f)={G}_0{\left(f/{f}_0\right)}^{\gamma}\left(1+i\eta \right)\Gamma \left(1-\gamma \right) \cos \left(\pi \gamma /2\right)+2\pi i\mu f. $$

This equation describes the cell’s complex viscoelastic modulus *G*(*f*) as a power law with exponent *γ*. The elastic (or storage) modulus of the cell, *G* ', is given by the real part of *G*(*f*), while the viscous (or loss) modulus *G* " is given by the imaginary part of the same equation. The parameters *G*_0_ and *f*_0_ are scale factors for the cell rigidity and frequency, respectively, and Γ(1 − *γ*) denotes the gamma function for the argument 1 − *γ*. The parameter *η* = tan(*πγ*/2) is the structural damping coefficient, while the term 2π*i*μ*f* represents Newtonian viscous damping, usually small except at high frequencies [16]. In the present work, we restrict ourselves to the frequency domain from 1 to 35 Hz, in which we can reasonably apply linear response theory. The structural damping terms in Eq. 1 follow [24] from Fourier-transforming the creep function, the time-dependent relation between strain and stress. Note that the corresponding damping ratio, given by *η*, is frequency-independent. It is usual to set the scale at *f*_0_ = 1 Hz, at which frequency *G*_0_ is of the order of the static Young’s modulus *E*. Indeed, for an incompressible medium [24], *G*_0_ = 3*E.*

The viscoelastic dynamic response of a cell is characterized only by the parameter γ, that has values ranging between 0 and 1. If *γ* → 0, the cell exhibits a solid-like behavior, while if *γ* → 1 a liquid-like behavior is approached [16, 17].

### Optical tweezers and the complex viscoelastic modulus of the cytoskeleton

Considering that the OT used to perform the experiment is at a fixed position, the viscoelastic properties of a chosen cell MCC immersed in the fluid within the culture chamber are obtained by submitting the microscope piezoelectric stage containing the sample to a forced oscillatory displacement described by ξ(*t*), with amplitude ξ_0_ and angular frequency ω = 2π*f*:

2 $$ \xi (t)={\xi}_0 \cos \left(\omega t\right). $$

**Fig. 1:**
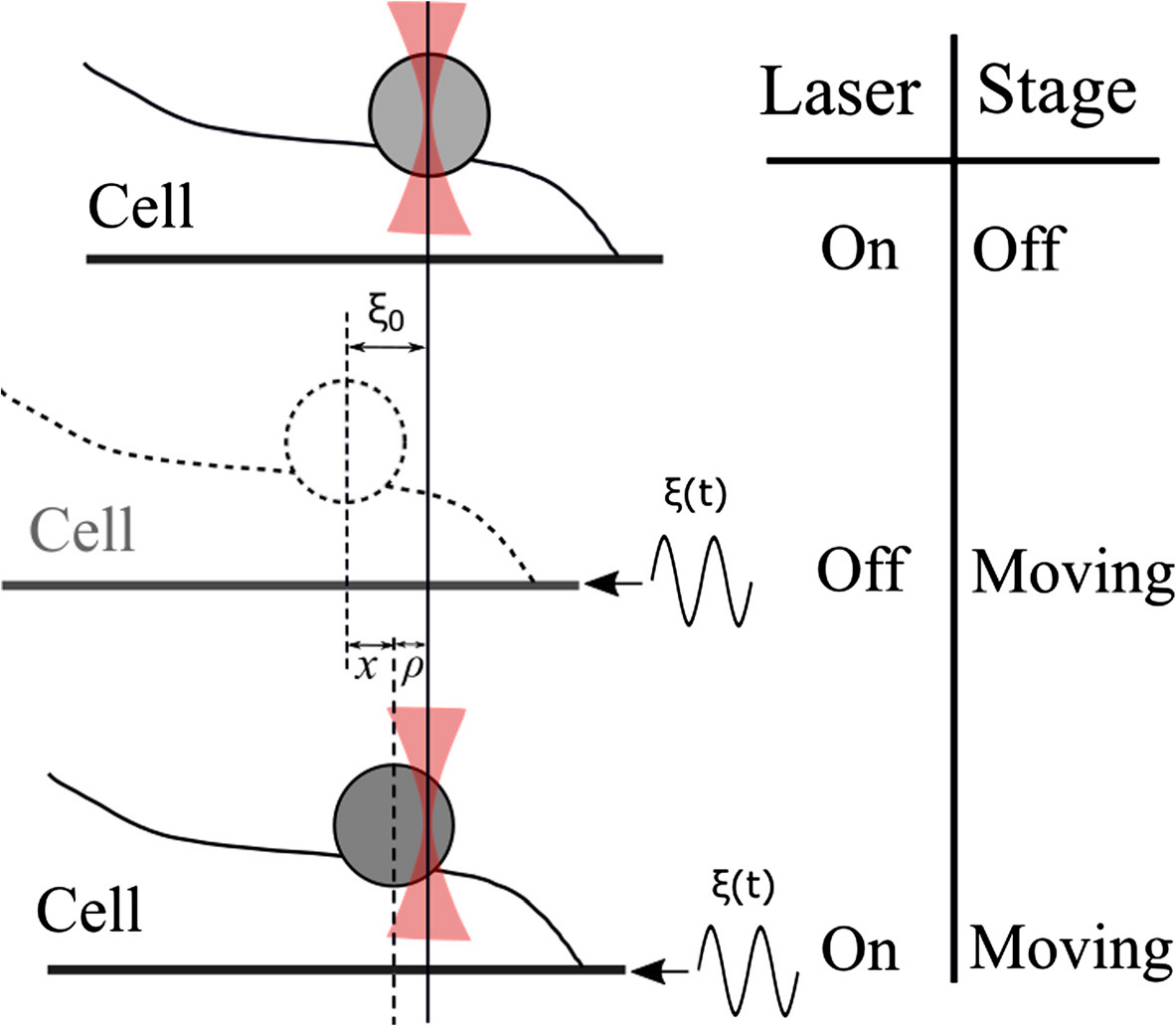
Definition of the variables in the cell rheology experiment. *ξ*(*t*) is the sample displacement, with amplitude ξ_0_, when an oscillatory movement is applied to the stage, with no optical forces acting on the bead, so that it just follows the stage displacement; *x* and *ρ* are the cell deformation and the bead displacement, respectively, when the laser is turned on and the stage is oscillating

A trapped uncoated bead is attached to the chosen spot on the cell surface by placing the bead in contact with it for about 5 s. The position *ρ*(*t*) of the trapped bead (Fig. 1) satisfies the equation of motion of a forced damped oscillator with negligible inertia:

3 $$ \beta \frac{d\rho }{dt}+\left(\kappa +{K}_c\right)\rho ={K}_c\xi +\beta \frac{d\xi }{dt}, $$

where *β* is the Stokes friction coefficient for the trapped bead [50], *κ* is the OT transverse stiffness, *ξ* is the sample displacement and *K*_*c*_ is the apparent cytoskeleton stiffness, which is related to the cell viscoelastic moduli.

In Eq. 3, the relation *x* = *ξ* − *ρ* between cell deformation *x*, sample displacement ξ and bead position *ρ* was used (Fig. 1). The Young’s modulus *E* of the cell is obtained from the apparent cytoskeleton stiffness *Κ*_*c*_, taking into account the geometrical details involved in the measurements, which are known to influence the results. In an optical tweezers study similar to the present one [24], the degree of immersion of the bead within the cell was taken into account. However, it has been found in other works [30, 48] that the results are additionally influenced by the underbead cell thickness *h*_*u*_, or rather, by its dimensionless ratio $\frac{h_u}{2a}$ to the bead diameter.

**Fig. 2:**
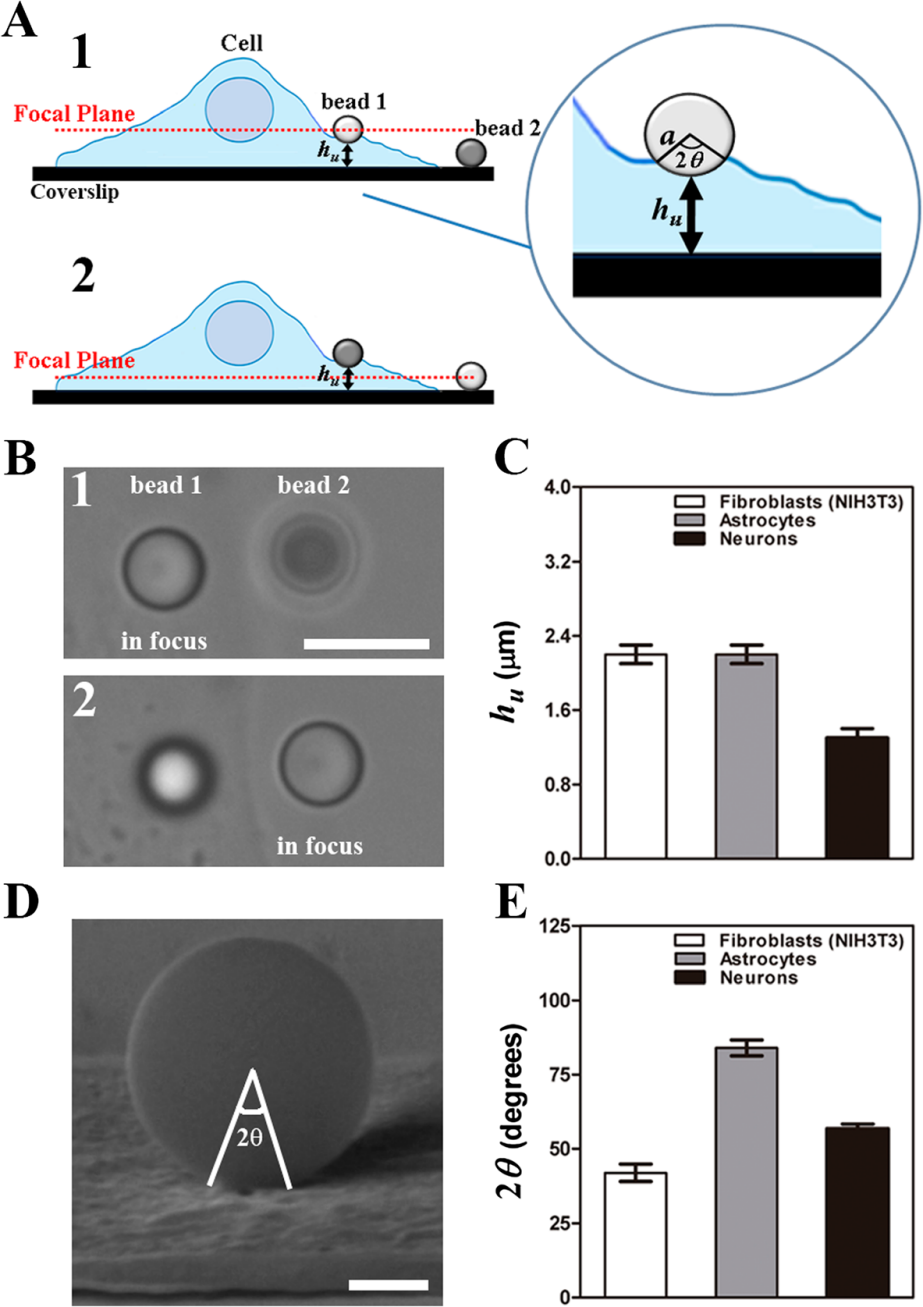
Measurements of *h*_*u*_ and *θ*. **a** Schematic representation of the experiment. The dashed lines represent the focused images in each case. The bead 1 is in contact with the cell and the bead 2 is attached to the coverslip. 2*θ* and *h*_*u*_ are both represented. **b** Images of both beads in the situations described in (**a**). Scale bar is 5μm. **c** Results for the under bead cell thickness *h*_*u*_. **d** Representative image of a bead attached to a fibroblast cell, showing the angle 2*θ*. Scale bar is 1μm. **e** Results for the cell-bead contact angle 2*θ*. Error bars in (**c**) and (**e**) represent the standard errors of the means

Following the approach described by Kamgoué and collaborators [30, 48], *Κ*_*c*_ can be written as:

4 $$ {K}_c=2\pi \alpha \left(\theta, \frac{h_u}{2a}\right)aE, $$

where $\alpha \left(\theta, \frac{h_u}{2a}\right)$ is a dimensionless purely geometrical function, assumed to be the same for all cell types. In Eq. 4, *h*_*u*_ represents the cell thickness below the sphere and the immersion angle *θ* defines the circular contact area between the cell surface and the bead (Fig. 2a). It is shown in Kamgoué’s work that taking into account the geometrical correction factor reduces the discrepancy between elastic parameters obtained by different methods.

In the frequency domain, according to the linear response theory and to the viscoelasticity correspondence (or equivalence) principle [51], Eq. 4 can be written in terms of the Fourier components of *ρ* and *ξ*. Since the factor $\alpha \left(\theta, \frac{h_u}{2a}\right)$ is purely geometrical, we follow [24] and replace *E* by the cell complex viscoelastic modulus *G*(*ω*) = *G* ' (*ω*) + *iG* " (*ω*), where *G* ' (*ω*) is the cell storage modulus and *G* " (*ω*) is the cell loss modulus. Assuming MCC incompressibility, only one elastic parameter characterizes the material (*E = 3G*_*S*_), where *G*_*S*_ is the cell complex shear viscoelastic modulus [51]. We have adopted Balland’s definition (*G = 3G*_*S*_) [24] in order to facilitate comparisons between our results and the results described in [24]. In this way the apparent complex cell stiffness *K*_C_^*^ = *K* ' + *iK* " is defined in terms of the cell complex viscoelastic modulus *G*(*ω*):

5 $$ {K}_c^{*}=2\pi\ \alpha \left(\theta, \frac{h_u}{2a}\right)aG\left(\omega \right). $$

In Eq. 5, the apparent storage cell stiffness is $K\hbox{'}=2\pi\;\alpha \left(\theta, \frac{h_u}{2a}\right)aG\hbox{'}$ and the apparent loss cell stiffness is $K\hbox{'}\hbox{'}=2\pi \alpha\ \left(\theta, \frac{h_u}{2a}\right)aG\hbox{'}\hbox{'}$.

By rewriting Eq. 3 in terms of complex Fourier expansions of the functions involved, we get:

6 $$ \beta \frac{d{\rho}^{*}}{dt}+\left(\kappa +{K}_C^{*}\right)\;{\rho}^{*}={K}_C^{*}\kern0.1em {\xi}^{*}+\beta \frac{d{\xi}^{*}}{dt}, $$

where

7 $$ {\xi}^{*}(t)={\xi}_0\kern0.1em {e}^{i\omega \kern0.1em t}, $$

and ρ^*^ is now a complex solution of the differential Eq. 6. The solution of the differential Eq. 3 is then given by:

8 $$ \rho (t)=\mathrm{R}\mathrm{e}\kern0.22em \left[{\rho}^{*}(t)\right]. $$

By substituting ρ*(*t*) = *Ae*^*iωt*^, and Eq. 7 into Eq. 6 we find.

9 $$ A=\frac{\xi_0\left({K}_C^{*}+i\omega \beta \right)}{\left(\kappa +{K}_C^{*}\right)+i\omega \beta }={\xi}_0\left[1-\frac{\kappa }{\sqrt{{\left(\kappa +{K}^{\prime}\right)}^2+{\left(\omega \beta +{K}^{{\prime\prime}}\right)}^2}}{e}^{-i\varphi}\right], $$

where

10 $$ \tan \varphi =\frac{\omega \beta +{K}^{{\prime\prime} }}{\kappa +{K}^{\prime }}. $$

The solution of the differential Eq. 3 gives the trapped bead position *ρ*(*t*), with the help of Eq. 8:

11 $$ \rho (t)={\xi}_0 \cos \left(\omega t\right)-\xi \hbox{'} \cos \left(\omega t-\varphi \right), $$

where

12 $$ {\xi}^{\prime }=\frac{\xi_0\kappa }{\sqrt{{\left(\kappa +{K}^{\prime}\right)}^2+{\left(\omega \beta +{K}^{\prime \prime}\right)}^2}}. $$

The parameters *ξ* ' and *φ* were experimentally determined for each frequency, as is described below in the Rheology Experiments section.

*K* ' and *K* " of the cell are then given by:

13 $$ K\hbox{'}=\kappa \left[\left(\frac{\upxi_0}{\upxi \hbox{'}}\right) \cos \upvarphi -1\right], $$

14 $$ K\hbox{'}\hbox{'}=\kappa \sin \upvarphi\ \left(\frac{\upxi_0}{\upxi \hbox{'}}\right)-\upomega \upbeta\ . $$

### Determination of the immersion angle *θ*

Polystyrene beads (*a* = 1.52 ± 0.02 μm) were attached to the surface of cells, for all tested cell types in this work. After the beads attachment, the cell cultures were immediately fixed with 2.5 % glutaraldehyde in 0.1 M cacodylate buffer (pH 7.4) for 40 min. They were then rinsed in the same buffer and dehydrated in ethanol. The samples were then dried using a critical point dryer (Leica Microsystems, Germany), gold-sputtered using a sputter coater (BAL-TEC, Liechtenstein) and observed in an EVO MA10 scanning electron microscope (Carl Zeiss, Germany). Images were acquired using SmartSEM software (Carl Zeiss). The angle 2*θ*, that defines the contact region of a bead of radius *a* with the cell surface, was measured for each cell type used in this work, following previously described procedures [52].

The cell-bead contact region (Fig. 2a) is defined by a circular area of radius *R*_*p*_ [52]:

15 $$ {R}_p=a\  \sin \theta . $$

### Determination of the under bead cell thickness *h*_*u*_

The parameter *h*_*u*_ was measured as the difference in height between two beads, one attached to the cell, at some distance from its edge [30], and another attached to the coverslip (Fig. 2a). The piezoelectric stage E-710 was set to move along the *z* direction with a controlled velocity *v* = 200 nm/s. One bead was kept in focus and the distance that the stage needed to scroll until the other bead reached the focus was measured. By comparing the gray level intensities as well as the outlines of both beads images, using ImageJ software (National Institutes of Health, Bethesda, MD), the value of *h*_*u*_ was determined. The entire process was recorded by a Digital Hamamatsu C11440-10C camera (Hamamatsu, Japan) with a frame rate of 100 fps.

### Geometrical function $\alpha \left(\theta, \frac{h_u}{2a}\right)$

An important parameter in the method described by Kamgoué and colaborators [30, 48] is the geometrical function $\alpha \left(\theta, \frac{h_u}{2a}\right)$, which is defined as:

16 $$ \alpha \left(\theta, \frac{h_u}{2a}\right)={A}_{\alpha}\left(\theta \right)+\frac{B_{\alpha}\left(\theta \right)}{h_u/2a}. $$

It is apparent from Eq. 16 that the underbead cell thickness correction is most important for small values of *h*_*u*_/2*a*, as expected, since it expresses the increasing influence of proximity to the rigid substrate.

The best fit to the coefficients in Eq. 16, obtained by an optimized finite element analysis, led to [30, 48]:

17 $$ {A}_{\alpha}\left(\theta \right)=2.321\times {10}^{-2}-2.054\times {10}^{-1}\theta +5.250\times {10}^{-1}{\theta}^2-1.338\times {10}^{-1}{\theta}^3, $$

18 $$ {B}_{\alpha}\left(\theta \right)=4.788\times {10}^{-3}-4.314\times {10}^{-2}\theta +1.020\times {10}^{-1}{\theta}^2-2.698\times {10}^{-2}{\theta}^3, $$

as polynomial functions of the immersion angle *θ*. The function $\alpha \left(\theta, \frac{h_u}{2a}\right)$ was determined for each cell type used in this work.

### Rheology experiments

**Fig. 3:**
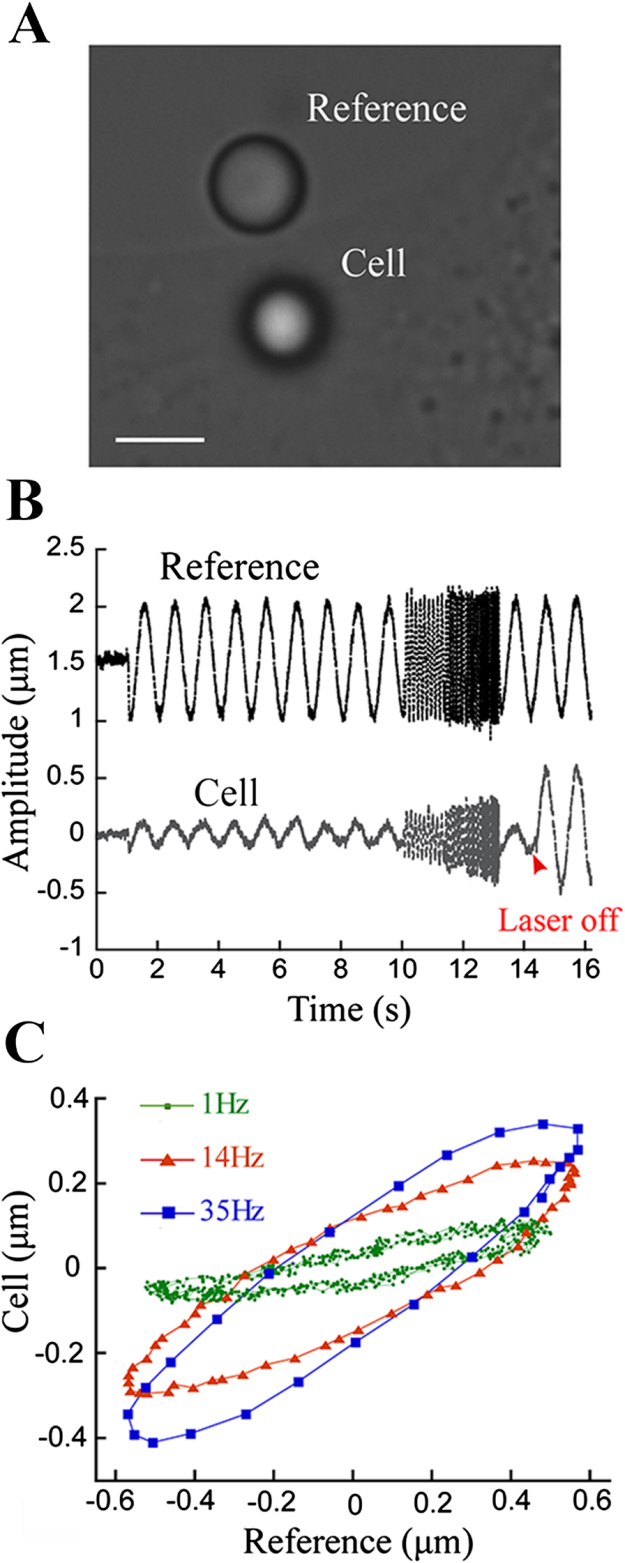
Rheology measurement of a fibroblast cell. **a** Representative image of the region of the cell where the measurement is performed. Both beads (“reference bead” and “cell bead”) are shown. Scale bar is 3 μm. **b** Plot of the amplitudes of both beads over time. A sinusoidal movement of 0.5 μm in amplitude is produced by the piezoelectric stage. The “reference bead”, oscillates following the movement of the piezoelectric stage. The “cell bead”, oscillates with a different amplitude and phase. Once the stage has oscillated covering the frequency range of interest, and before the oscillation has stopped, the laser is turned off in order to verify if the “cell bead” is still in contact with the cell, following the same oscillating movement as the “reference bead”. **c** Plot of “cell bead” vs “reference bead” displacements for 1, 14 and 35Hz corresponding to the oscillations in (**b**)

In order to reduce the mechanical variability of the parameters found in different regions of cells [27, 31, 45], the measurements were always performed at similar distances from the cell edge (Fig. 2b) for fibroblasts and astrocytes. For neurons, we limited the probe to the neurite region. An uncoated polystyrene bead of radius *a* = (1.52 ± 0.02)*μm* (named “reference bead” in Fig. 3a) was attached to the sample chamber glass bottom, in a region near the chosen cell. Another uncoated polystyrene bead of same radius (named “cell bead” in Fig. 3a) was trapped by the OT and attached to the cell surface by pressing it against the cell for about 5 s and then returning to its axial equilibrium position in the trap before starting the measurement. The microscope piezoelectric stage, and not the laser, is the one that was moved, by submitting it to different sinusoidal displacements of amplitude *ξ*_0_ = (0.500 ± 0.001) *µ*m and frequencies (*f*) varying from 1 Hz to 35 Hz. The displacements of both beads were recorded simultaneously by a Digital Hamamatsu C11440-10C camera (Hamamatsu, Japan) at a frame rate of 800 fps. After covering the frequency range of interest and before the oscillation had stopped, the laser was turned off, in order to verify that the “cell bead” was still attached to the cell. For each experiment, the coverslip position and the cell response were always determined by the “reference bead” and “cell bead” displacements.

Knowing the “reference bead” and “cell bead” positions as functions of time, we used Eqs. 2 and 11 to fit the oscillatory response of both beads, respectively. From the curve fitting we obtained the amplitude *ξ* ' and the phase *φ*. Then from Eqs. 13 to 14 we determined the apparent storage *K* ' and loss *K* " cell responses of a chosen cell as functions of frequency. The elastic *G* ' (*ω*) and viscous *G* " (*ω*) moduli of cells were obtained from Eq. 5, taking into account the geometrical function $\alpha \left(\theta, \frac{h_u}{2a}\right)$. Finally, fitting the real part of Eq. 1 to *G* ' (*ω*) and its imaginary part to *G* " (*ω*) for a chosen value of *f*_0_, we obtained the parameters γ, *G*_0_ and μ, previously defined. The data were analyzed using ImageJ and Kaleidagraph (Synergy Software, Essex Junction, VT, USA) softwares.

## Results and discussion

### Geometrical parameters for fibroblasts, astrocytes and neurons

We applied OT to dynamically perturb and measure the viscoelastic MCC response of fibroblasts, neurons and astrocyte in the frequency range from 1 to 35 Hz (Fig. 1). The viscoelastic moduli *G* ' (*ω*) and *G* " (*ω*) were obtained from Eqs. 5, 13 and 14, employing the measured values of *h*_*u*_ and *θ* (see the Methods section). The values of the under bead cell thickness *h*_*u*_ (Fig. 2c) were measured following the procedures illustrated in Fig. 2a and 2b. The values of cell-bead immersion angle *θ* (Fig. 2e) were measured as shown in Fig. 2d. The results obtained for each cell type employed in this work are presented in Table 1. From *h*_*u*_ and *θ*, the value of $\alpha \left(\theta, \frac{h_u}{2a}\right)$ was calculated using Eq. 16 (see the Methods section).

**Table 1:** Summary of the experimental values for the under bead cell thickness *h*_*u*_ and the contact angle *θ*. From these experimental values the geometrical factor *α*(*θ*, *h*_*u*_/2*a*) was calculated using Eq. 16

Cell Type	*h*_*u*_ (μm)	*θ* (°)	*α*(*θ*, *h*_*u*_/2*a*)
Fibroblasts (NIH3T3)	2.2 ± 0.1	20 ± 2	0.011 ± 0.005
Astrocytes	2.2 ± 0.1	42 ± 2	0.13 ± 0.02
Neurons	1.3 ± 0.1	28 ± 1	0.044 ± 0.005

The fibroblasts and astrocytes cell heights near their edges have similar values, 2.2 ± 0.1 μm, almost twice the value obtained for neurons, 1.3 ± 0.1 μm, (Fig. 2c and Table 1). The highest value observed for the bead immersion angle *θ* was found for astrocytes (Fig. 2e and Table 1). This result already suggests that the astrocyte MCC is softer when compared to neurons and fibroblasts, since, by using the same pressing force with the optical tweezers, the trapped bead penetrates deeper in astrocytes.

### Viscoelastic moduli for fibroblasts, astrocytes and neurons

Figure 3 shows the MCC response of a fibroblast cell to an oscillatory perturbation varying from 1Hz to 35Hz. The bead attached to the coverslip, denoted as “reference bead”, oscillates with the same amplitude and in phase with the microscope stage. However, the bead attached to the cell surface and trapped by the OT, denoted as “cell bead”, oscillates with a decreased amplitude and, although this is not obvious from Fig. 3b, it has a shift in phase when compared to the microscope stage movement (Fig. 3c). By evaluating the differences in amplitude and phase between both beads from the curve fitting of Eqs. 2 and 11 (see methods section), we were able to determine the MCC viscoelastic moduli for the three cell types used in this work. For comparative purposes, plots of *K* ' *vs θ* and *K* " *vs θ* were presented (Additional file 1: Figure S1).

**Fig. 4:**
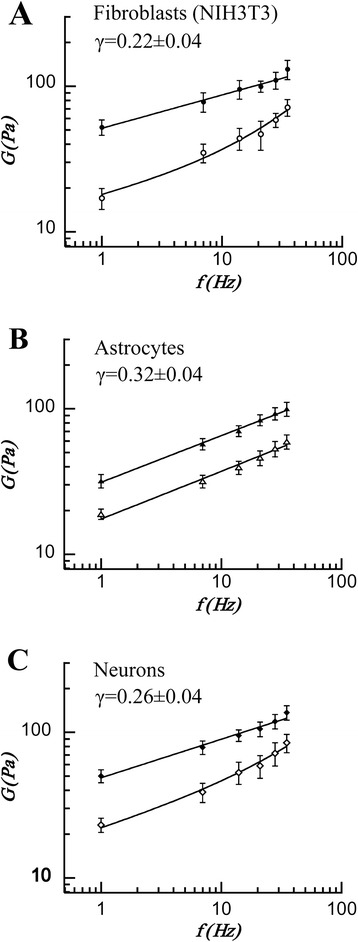
The average *G* moduli as a function of frequency *f* varying from 1 to 35Hz for (**a**) fibroblasts *G* ' –●, *G* " –○; (**b**) astrocytes *G* ' –▲, *G* " –Δ and (**c**) neurons *G* ' –♦, *G* " –◊. The experimental values were fitted using the structural damping law (solid line). *G* ' and *G* " both grow according to a power law with exponents *γ* = 0.22 ± 0.04 for fibroblasts, *γ* = 0.26 ± 0.04 for neurons and *γ* = 0.32 ± 0.04 for astrocytes

The results for the viscoelastic moduli of fibroblasts, astrocytes and neurons are shown in Fig. 4. The error bars in the graphs represent the standard errors of the mean values obtained for at least 20 different cells.

The values of *G*_0_, *γ* and μ (Table 2) were obtained by fitting Eq. 1 (see the Methods section) to the experimental data of *G* ' and *G* ". The value *f* = 1Hz was chosen to set the scale frequency of the data. The fits in Fig. 4 show good agreement with the soft glassy rheology model and the structural damping law within the frequency range of our measurements. While the fit to a power law over our frequency range might be questioned, its justification resides in the very good fit obtained and in our employment of the widely adopted soft glass rheology model within the linear response range and the physiological normal metabolic cell domain.

**Table 2:** Parameters obtained from the rheology experiments performed on fibroblasts, astrocytes and neurons. The results were fitted with Eq. 1, setting the scale frequency *f*_0_ = 1 Hz

Cell type	*G*_0_ (Pa)	*γ* (dimensionless)	*μ* (Pa · s)
Fibroblasts (NIH3T3)	55 ± 5	0.22 ± 0.04	0.15 ± 0.05
Astrocytes	36 ± 2	0.32 ± 0.04	0.00 ± 0.06
Neurons	53 ± 4	0.26 ± 0.04	0.09 ± 0.06

For comparisons with viscoelasticity results previously reported in the literature within a comparable frequency range, it suffices to consider the parameters of Table 2. To the best of our knowledge, our measurements for neurons and astrocytes are the first ones performed with optical tweezers. A table of Young’s modulus measured by AFM for many cell types is given by Kuznetsova and collaborators [53].

For the three cell types used in this work, *G* ' and *G* " both increased with the frequency. This behavior has also been reported for other cell types [16, 17, 22, 47, 54]. The viscoelastic response of fibroblasts followed a power law with exponent *γ* = 0.22 ± 0.04. The neurons’ viscoelastic behavior was fitted by an exponent *γ* = 0.26 ± 0.04. The highest value for the exponent was obtained for astrocytes (*γ* = 0.32 ± 0.04). All γ values are in good agreement with the range of values reported in the literature, from 0.1 to 0.3, regardless of the technique employed [10, 14, 17, 24, 26, 32].

For NIH3T3 fibroblasts, the absolute values found for *G* ' and *G* " were in the ranges (56 ≤ *G* ' ≤ 139) Pa and (18 ≤ *G* " ≤ 76) Pa, respectively. Although the viscoelastic moduli values reported in the literature for these cells show wide variation, ranging from a few Pa to kPa [10, 25–27, 29, 31, 33, 34, 44], the values obtained in this work are of the same order of magnitude as those reported in recent literature using OT [32] and even using another technique (AFM) [26, 31, 32]. Very recently, an innovative technique (rotational magnetic spectroscopy) also led to values with order of magnitude comparable to ours [55].

On the other hand, neurons exhibit an elastic modulus (50 ≤ *G* ' ≤ 136) Pa, and a viscous modulus (23 ≤ *G* " ≤ 84) Pa, while for astrocytes the elastic modulus is in the range (32 ≤ *G* ' ≤ 100) Pa and the viscous modulus in the range (19 ≤ *G* " ≤ 59) Pa.

Our results for neurons are of the same order of magnitude as those found in some previous studies [36, 47, 56–58]. For astrocytes, the existing results range from hundreds of Pa [47] to kPa [13, 59, 60], different from what we found (tens of Pa). It has been shown that the viscoelastic properties of astrocytes are strongly dependent on the cell cytoskeleton maturation state [46, 60]. Even though the absolute values that we find for astrocytes are different from those found in literature, we observe that the astrocyte cytoskeleton is softer in comparison with fibroblasts and neurons, in agreement with previously reported data [47].

According to Table 2, fibroblasts exhibited not only the highest elastic modulus but also the most solid-like behavior. This may be related to their commitment to provide structural support within connective tissue and with their fibrous nature, mainly composed of actin stress fibers.

Astrocytes present a lower elastic modulus, almost half those obtained for fibroblasts. Astrocytes are pointed out as the most abundant glial cell in the central nervous system and, like fibroblasts, they also have a structural and mechanical supporting function. However, the soft nature of astrocytes, measured in this work and also previously probed with other techniques [47], may provide a more appropriate support for neurons, since the latter prefer softer substrates to proliferate [61] and differentiate [6]. The role of astrocytes has been compared to that of cushioning materials in packaging [43]. The rheology results for astrocytes are in agreement with the adhesion angle measurements, which present the deepest bead insertion, as expected from their softer nature.

Neurons have also been characterized as soft cells [58]. They are able to change their mechanical properties during neuronal growth and development [36]. The values of γ in Table 2 indicate that they exhibit a more liquid-like behavior than fibroblasts but a more solid-like behavior when compared to astrocytes.

The Newtonian viscous damping coefficient μ obtained is relatively small for the three cell types used in this work. According to previous studies, this Newtonian viscosity is expected to be small at low frequencies [16]. In our case we explored the viscoelastic properties of cells up to 35Hz, which could be considered a low frequency in comparison with frequencies that other techniques have accessed (100–1000 Hz) [16, 35, 47]. Magnetic twisting cytometry, in particular, covers from much lower to much higher frequencies than the present study [62]. Since the values of μ, as well as *G*_0_, depend on the cell-bead geometry [16], a direct comparison with other reported values of μ would not be feasible. However, techniques that use the structural damping model and that have explored the viscoelastic behavior of NIH3T3 fibroblasts up to 200 Hz still found small values of μ [26, 31]. For astrocytes and neurons, μ values are not reported.

## Conclusions

This work applies an optical tweezers-based methodology to measure the viscoelastic properties of the MCC, taking into account all relevant parameters that affect the cell’s viscoelastic moduli [30, 48]. Discrepancies with previous results remain, some of them inevitable, given the natural variability of cells. However, we carefully evaluate the under bead cell thickness and the cell-bead immersion angle, two important parameters that can change dramatically the viscoelastic moduli obtained from the experimental analysis. Our results support the evidence that the MCC viscoelastic moduli should be in the range of tens to hundreds of Pa. We apply the developed methodology to three cell types: fibroblasts, neurons and astrocytes. We find that fibroblasts and neurons present similar viscoelastic properties, both stiffer than astrocytes. The methodology described in this paper can be used to investigate structural and mechanical cell processes, such as cytoskeleton rearrangement during cell migration and phagocytosis. In addition, it can also be used to study other kinds of biological materials that exhibit similar viscoelastic behaviors.

## Availability of supporting data

All the supporting data are included as Additional file 1.

## Abbreviations

AFM, atomic force microscopy; EDTA, ethylenediaminetetraacetic acid; MCC, membrane-cortex complex; OT, optical tweezers, MTC, magnetic twisting cytometry; PBS, phosphate buffered saline; SGM, soft glassy material

## Additional file

Additional file 1: Figure S1. Plots of *K* ' and *K* " measured for fibroblasts, neurons, and astrocytes vs immersion angle θ for the different frequencies probed in this work. (DOCX 110 kb)
