# Supplementary material for: Rheological properties of cells measured by optical tweezers
Source: BMC Biophys. 2016 Jun 22;9:5. doi: 10.1186/s13628-016-0031-4 (PMC4917937; doi:10.1186/s13628-016-0031-4)
Supplement: Additional file 1: Figure S1. — Plots of K ' and K " measured for fibroblasts, neurons, and astrocytes vs immersion angle θ for the different frequencies probed in this work. (DOCX 110 kb) [file 13628_2016_31_MOESM1_ESM.docx]

**Additional file 1 for:**

**Rheological Properties of Cells Measured by Optical Tweezers**

Yareni A. Ayala^1,2,§^, Bruno Pontes^1,§^, Diney S. Ether^1,2^, Luis B. Pires^1,2^, Glauber R. de S. Araujo^3^, Susana Frases^3^, Luciana F. Romão^4^, Marcos Farina^1^, Vivaldo Moura-Neto^5^, Nathan B. Viana^1^*^,^*^2,*^ and H. Moysés Nussenzveig^1,2^

^1^LPO-COPEA, Instituto de Ciências Biomédicas, Universidade Federal do Rio de Janeiro, RJ, 21941-902, Brazil.

^2^Instituto de Física, Universidade Federal do Rio de Janeiro, RJ, 21941-972, Brazil.

^3^Laboratório de Ultraestrutura Celular Hertha Meyer, Instituto de Biofisica Carlos Chagas Filho, Universidade Federal do Rio de Janeiro, RJ, 21941-902, Brazil.

^4^Universidade Federal do Rio de Janeiro – Pólo de Xerém, Duque de Caxias, RJ, 25245-390, Brazil.

^5^Instituto Estadual do Cérebro Paulo Niemeyer, Rio de Janeiro, RJ, 20231-092, Brazil.

* Corresponding author

^§^ Yareni Ayala and Bruno Pontes contributed equally to this work.


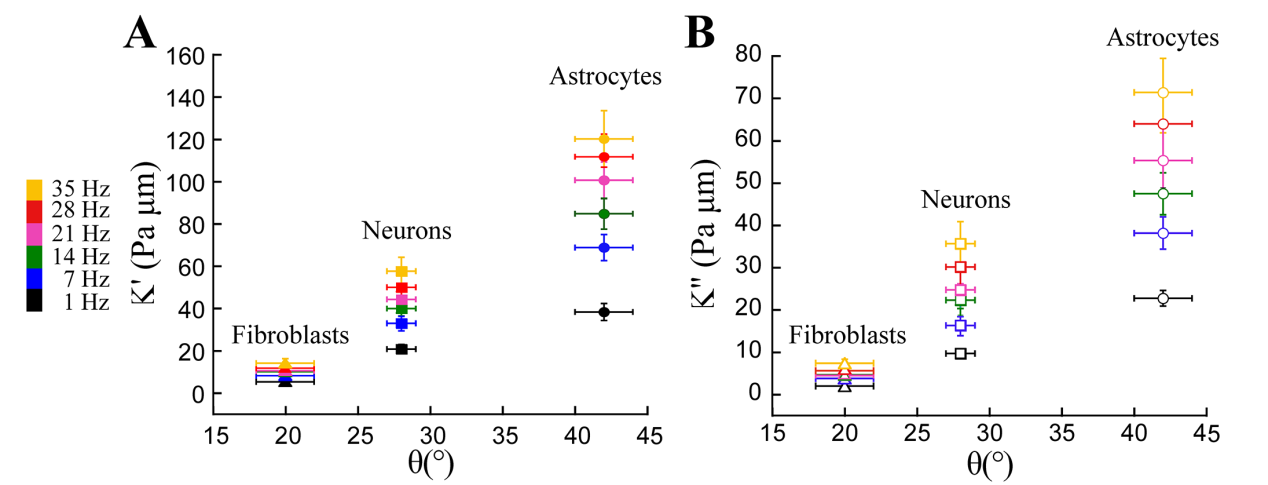


**Figure S1**: (A) Plot of $K^{'}$ $K_{C}^{*}=K^{'}+iK^{''}$ values measured for fibroblasts, neurons, and astrocytes *vs* immersion angle *θ* for the different frequencies probed in this work. (B) Similar plot for $K^{''}$ values. All error bars represent the standard error of the means.
